# Co‐location, an enabler for service integration? Lessons from an evaluation of integrated community care teams in East London

**DOI:** 10.1111/hsc.13211

**Published:** 2020-11-05

**Authors:** Mirza Lalani, Martin Marshall

**Affiliations:** ^1^ London School of Hygiene and Tropical Medicine London UK; ^2^ University College London Manchester London UK

**Keywords:** co‐location, integrated care, partnership working

## Abstract

In an attempt to support care integration that promotes joined up service provision and patient‐centred care across care boundaries, local health and social care organisations have embarked on several initiatives and approaches. A key component of service integration is the co‐location of different professional groups. In this study, we consider the extent to which co‐location is an enabler for service integration by examining multi‐professional community care teams. The study presents findings from a qualitative evaluation of integrated care initiatives in a borough of East London, England, undertaken between 2017 and 2018. The evaluation employed a participatory approach, the Researcher‐in‐Residence model. Participant observation (*n* = 80 hr) and both semi‐structured individual (*n* = 16) and group interviews (six groups, *n* = 17 participants) were carried out. Thematic analysis of the data was undertaken. The findings show that co‐location can be an effective enabler for service integration providing a basis for joint working, fostering improved communication and information sharing if conditions such as shared information systems and professional cultures (shared beliefs and values) are met. Organisations must consider the potential barriers to service integration such as differing professional identity, limited understanding of roles and responsibilities and a lack of continuity in personnel. Co‐location remains an important facet in the development of multi‐professional teams and local service integration arrangements, but as yet, has not been widely acknowledged as a priority in care practice. Organisations that are committed to greying care boundaries and providing joined up patient care must ensure that sufficient focus is provided at the service delivery level and not assume that decades of silo working in health and social care and strong professional cultures will be resolved by co‐location.

**What is known about this topic and what this paper adds?**

- Integrated care is considered an important principle for organising the delivery of care services and at the service delivery level, provider organisations have sought to promote integration through the development of multi‐professional teams that are co‐located
- In this study, co‐location aided the development of working and social relationships and facilitated communication and information sharing in multi‐professional teams
- However, co‐location of health and social care professionals alone is not an effective enough enabler for service integration
- Organisations must consider addressing several issues such as differing professional cultures, limited continuity of care and a lack of joint patient/client record for health and social care

## 1 BACKGROUND

Across the United Kingdom, health and care systems are having to respond to substantial demands due to an ageing population with complex care needs and multi‐morbidities set against the backdrop of constrained financial resources (Ham et al., 2011). It has been suggested that care integration could play an important role in addressing these challenges (Humphries, 2015). Since the introduction of the Health and Social Care Act in 2012 in England, momentum for integrated care has gathered pace (Richardson, 2013). While the reconfiguration of health and social care at the system level has garnered much attention, there has also been significant change at the level at which services are delivered (Ham, 2018). In 2019, the NHS Long Term Plan in England was accompanied by the mandating of Primary Care Networks whereby primary care practices would have to work together as well as with community, mental health, social care, acute care and voluntary services in their local areas (Baird, 2019). A proposed benefit of such an approach is to ensure that care provision is tailored to the local population.

In an attempt to support service integration that promotes joined up, seamless service provision and patient‐centred care across care boundaries, local health and social care organisations have embarked on several different approaches and initiatives since 2012, with a predominant focus on coordination between different professional groups and case management (Ham & Murray, 2015; Turner et al., 2016). The co‐location of different care professionals has been identified as a key component of service integration (Kaehne & Catherall, 2012). Co‐location refers to different professional groups situated in the same workspace. Co‐location outside of a hospital setting is certainly not a new phenomenon. In the United Kingdom, for many years, General Practice (GP) surgeries hosted GPs, nurses (community and practice), administrative and clerical staff (Memon & Kinder, 2017). More recently, the professional groups based in primary care facilities have diversified further to include health visitors, pharmacists, social prescribers, mental health workers and social workers (Bonciani et al., 2018; Kharicha et al., 2005). The recent trend in centralisation of community nursing services with services being procured by community health providers has created a community nursing care provision that is located away from primary care (Lalani et al., 2019). Community nursing is the principal leader in community care provision but other professionals such as therapists, mental health and sometimes social workers also feature, often as part of a multi‐professional team.

Co‐location is thought to promote collaborative working, enable effective communication, develop more prosperous working and social relationships, overcome issues of professional culture and ultimately may have a positive impact on patient outcomes (Cameron & Lart, 2003). Indeed, as co‐located professionals have more frequent formal and informal opportunities to meet and share information, they are more likely to reach consensus in decision making, which enhances clinical practice (Bonciani et al., 2018). Moreover, co‐location may foster service innovations through professionals learning from each other and drawing upon their diverse experiences and skills sets, although in the main, innovations arise from an attempt to delivery more efficient and effective services (Memon & Kinder, 2017). Where partnership working has encountered challenges, it is often due to organisations failing to address issues of organisational and professional cultures, infrastructure problems such as IT systems and equity in the resources provided (Christiansen & Roberts, 2005; Hudson, 2002).

An often referred to success of co‐location outside of the hospital setting is the situating of mental health professionals within primary care which has reportedly improved the quality of care and access to patients with mental health illness (Williams et al., 2006). This is as a result of more frequent opportunities for GPs and mental health workers to hold face‐face discussions on aspects of patient care. Co‐location has enabled mental health workers to facilitate the understanding of GPs with regard to referral pathways into secondary mental healthcare, reducing administrative burden. Furthermore, providing mental health workers access to online GP patient records assists in the sharing of information in a more convenient and timely manner (NHSE, 2018).

In this study we consider the role of co‐location in service integration in community care in Tower Hamlets, a borough of East London, England. We aimed to assess the extent to which co‐location was an enabler for service integration among multi‐professional teams in community care.

## 2 METHODS

### 2.1 Subjects and Settings

The study presents findings from a qualitative evaluation of integrated care initiatives in Tower Hamlets, East London. In 2015, a partnership of multi‐speciality community provider organisations was awarded ‘Vanguard’ status (support and funding to develop innovative models of care which other parts of the country can learn from) by the arms‐length body of the Department of Health and Social Care, NHS England. The partnership comprised a collaboration of health and social care commissioners and providers as well as the local Council for Voluntary Services. The Vanguard sites were awarded substantial funding to further develop local integrated care approaches with a primary focus on complex care provision (THT, 2018).

The borough is comprised of four localities (population of 60,000–80,000) and each locality has a multi‐professional community care team known as an Extended Primary Care Team (EPCT) which provides community nursing and therapies for patients aged over 18 and resident in the borough. The north‐west and north‐east EPCTs were co‐located on the same floor of a building in central Tower Hamlets. The latter moved location from a primary care centre during the course of the evaluation. The south‐west and south‐east teams were located within their own localities with the latter based in a primary care centre. The development of EPCTs and their co‐location was a key deliverable of the Community Health Services contract awarded to the Tower Hamlets Alliance Partnership in 2016. Prior to this, the extent to which health professionals in the community were co‐located varied. Indeed, at the commencement of the evaluation, the therapists and community nurses from the NE team were based in different locations.

The teams provide care coordination and case management for patients whose needs are most appropriately met by co‐located community care professionals; community/district nurses, community health care assistants, occupational therapists, physiotherapists, mental health nurses, rehabilitation support workers and care navigators (care navigators provide non‐clinical support to patients pertaining to a wider variety of aspects of health and social care; HEE, 2016). At the time of the study, each of the EPCTs was supported by a social worker from the Local Authority, although this was sporadic. Social workers were not permanently co‐located with the EPCTs but were expected to visit frequently for case discussions as well as attend EPCT monthly meetings. Social worker attendance at the EPCT offices varied from once a fortnight to three times a week.

### 2.2 Study design

The study commenced in May 2017 and was completed in November 2018. In this study, we draw on the findings from field notes of observations and semi‐structured interviews (individual and group interviews) with stakeholders from the EPCTs operating in community care. Interviews were conducted by ML, a researcher with experience of conducting health services research using qualitative methods.

The evaluation used the Researcher in Residence model, a participatory approach to research. Local health and care partners expressed an interest in this model, as they were keen to develop their programme in response to locally generated and co‐created evidence in ‘real‐time’ (Lalani, 2018). The lead researcher (ML) was embedded in the Vanguard programme in Tower Hamlets. In response to a recognised concern that ‘established approaches to getting health services research into practice are not radically changing the extent to which management decisions are influenced by scientific evidence,’ the Researcher in Residence model embraces the concept of ‘co‐creating’ knowledge between researchers and practitioners (Gradinger et al., 2019; Lalani et al., 2019; Marshall et al., 2014). The model placed the researcher as a key member of the delivery team within the organisations under study, as opposed to an external observer of change. ML co‐created knowledge with participants in the study; an evaluation steering group was set up involving stakeholders from health and social care organisations to co‐design the research protocols and discuss the key findings from the research. The participatory approach facilitated the mobilisation of existing knowledge (from the academic and policy literature) and newly created evidence (generated by the research) across the localities and, to an extent, influenced implementation and development of community care service provision locally.

### 2.3 Data collection

We conducted 16 semi‐structured individual and six group interviews (total *n* = 17 EPCT staff) and participant observation of relevant meetings amounting to approximately 80 hr. Interviews were undertaken with middle managers, service managers, EPCT leads, health professionals from the teams as well as social workers aligned to the EPCTs (see Table 1). We used a purposive sampling strategy to identify relevant middle and service managers from both health and social care. We also interviewed the four EPCT team leads. We purposefully selected a range of EPCT staff for group interview considering their level of experience, qualification and profession. We interviewed staff on permanent contracts with a provider organisation and agency workers. Given the four teams comprised over 100 staff, working different shift patterns, group interviews were determined as the most efficient approach to obtain the potential breadth and depth of views among EPCT professionals. Given the embedded approach to the evaluation, most participants were known to the researchers.

**TABLE 1:** Participant information with professional roles

Middle managers	Service managers	Nurses	Therapists	Care navigators	Social Workers
3 (2 from the healthcare provider, 1 from the Local Authority)	4 service managers (2 from health and 2 from social care)	9 community/district nurses (including 2 team leads) 2 mental health nurses	7 (including 2 team leads and 2 rehabilitation support workers)	4 care navigators	4 (including 2 team leads)

Interview guides were formulated using relevant themes from the literature on models of integrated care and locality based approaches to partnership working and implementation. In addition, interview guides were informed by data from participant observation. An inductive approach was taken with emerging themes from initial interviews used as a basis for further iterations of the interview guide. The interviews covered broad topic areas such as: (a) understanding of the Vanguard programme's purpose; (b) the development of the locality integrated care model and perceived impacts of the programme on staff and service users and expectations on the future development of the model to meet the needs of the local population; and (c) the facilitators and barriers to partnership working. Interviews with staff were held at the participant's workplace in a private meeting room. Interviews lasted between 45 and 75 min.

### 2.4 Data analysis

Interviews were audio‐recorded and transcribed verbatim. Data were managed using NVivo version 11.0. ML conducted qualitative analysis using a thematic framework approach to code the data and identify patterns and themes (Green & Thorogood, 2018). The framework was developed inductively from the data with a focus on the theme of co‐location. Data were also informed by field notes from participant observation. Components of the analysis plan, including the review of the coding framework and co‐interpretation of the findings, were undertaken by both authors.

### 2.5 Ethics

Ethics and governance approvals were provided by the NHS Research Ethics Committee (REC ref. 17/SC/0687) and the Health Regulatory Authority. All interview participants were approached by email by the researcher who outlined the purpose of the study and interview process where appropriate. Written informed consent was obtained from each participant prior to interview. Participants agreeing to interview returned their signed consent forms at the time of the interview. Participants were assured of confidentiality and anonymity and that participation was voluntary, and that they were free to withdraw from the study. No participants withdrew their consent.

## 3 RESULTS

The data relevant to co‐location are drawn from a larger dataset which was part of the aforementioned evaluation of the Tower Hamlets together programme (Lalani, 2018). Co‐location emerged as a prominent theme in the project. Here, we present our findings pertinent to co‐located teams in community care. Several themes associated with co‐location emerged from the data and acted as both barriers and enablers to service integration. The findings are organised under two main themes; structural and relational aspects of co‐location.

### 3.1 Structural aspects

#### 3.1.1 Infrastructure

Extended Primary Care Team staff mentioned that co‐location had been accompanied by an improvement in the quality of facilities including more office and social space (staffrooms), and upgrades in the IT infrastructure. Indeed, the provision of additional space was planned with a view to social workers being permanently situated with the EPCT teams. Yet, social workers commented that the quality of space was often an issue when visiting the EPCT teams. Furthermore, it was acknowledged that a lack of a shared patient/client record with community health and social care using different IT systems had been a notable barrier to effective integration. Managers hoped that co‐location would at least facilitate access for social workers to patient health records, although they conceded that this was an area of development that needed to be addressed if the benefits of service integration were to be realised.

…organisations have different data systems that's a major problem. I was working with one person who was delivering care to very vulnerable individuals and was having to fill in three databases with the same information, which was just ridiculous. The stuff that they were doing for health, the stuff they were doing for social care, and the stuff they were doing for their own organisation, there wasn't a way of kind of simplifying (Middle manager).

Soon after the study commenced, community health patient records were integrated with primary care. This development was important in light of three of the EPCTs having been relocated away from GP surgeries, thereby minimising opportunities for face‐face discussion with GPs, an aspect that most staff valued highly.

### 3.2 Service design

Middle and service managers responsible for the EPCTs as well team members remarked on the merits of the design of the EPCT service and how co‐location was integral to optimising service delivery. The advantages mentioned included more straightforward and rapid intra‐team referrals and a reduction in duplication of care (e.g. repetition of taking medical history from patients). Co‐location was thought to reduce bureaucracy and, hence, promote time efficiency in service delivery. In turn, it was suggested that this may improve the patient experience with the scope for joint visits, limiting the number of appointments, thereby minimising inconvenience to patients.

Last week when I visited a patient I noticed she needed physiotherapy input, because the daughter complained to me that she has got right sided weakness. So I came and spoke to one of the physios and I said this is what I found and observed and this is what the daughter is saying; what do you think? And then he made an arrangement with the daughter to visit the next day. The referral was much quicker, there was no waiting (EPCT team member).

However, at the time of the study, the community health service was undergoing a significant system restructure. This resulted in some redundancies of permanent staff, a change in the approach to triaging of patients (which was perceived to have increased administrative work) and a feeling among existing staff that the restructure had been undertaken without the appropriate consultation of the affected teams. Some EPCT staff believed that many of the benefits of co‐location were nullified by the uncertainty caused to staff during the course of the restructure.

### 3.3 Relational aspects

#### 3.3.1 Information sharing and communication

Middle and service managers mentioned that co‐location had facilitated more frequent opportunities to share information, enhancing communication. The sharing of information was both formal and informal. Co‐location had enabled ‘corridor conversations’ about patients. These opportunities for informal case discussion were highly valued by the EPCT staff because strategies for addressing issues in care were actioned more swiftly thereby circumventing some of the more formal lengthy approaches to sharing information. In a few cases, informal discussions prompted joint visits to patients thereby enhancing care coordination.

I think there is a bit of rediscovering in primary care and community care, as there is a bit of rediscovering in secondary care that collaborative working with nursing, allied professionals, Social Services in delivering the best possible outcome to a patient involves talking to each other and co‐location helps with this (Service manager).

However, as social workers were not permanently based with the EPCTs, this limited the effectiveness of information sharing and communication between health and social care. Most opportunities for information sharing were through formal arrangements such as meetings. Yet, it was observed that, despite being invited, social workers seldom attended the monthly EPCT business meetings where major updates on local health service developments were shared. This was attributed to the frequent turnover of social work staff. One of the EPCT leads held fortnightly complex care meetings where patients with the most severe care problems were discussed. EPCT staff described these meetings as ‘MDTs without GPs.’ A social worker regularly attended this meeting which was held at the EPCT offices and they took advantage of the opportunity by spending the remainder of the day working with the EPCT staff.

Social workers also mentioned that quite often if they required information on a service user they would contact the care navigator who were seen by both health and social care professionals as a conduit between the two sectors through which information was shared and communication optimised.

#### 3.3.2 Understanding of roles and responsibilities

Co‐location enabled health professionals to develop a better understanding of each other's roles and responsibilities. The EPCT staff mentioned that co‐location had fostered joint training as well as formal and informal learning. This was particularly useful when undertaking case triage, as therapists, nurses and care navigators were able to jointly consider a care plan for a patient and share out the responsibilities of care provision minimising duplication.

Training together as a group means people are more aware of what other people are doing…people are sort of learning more from each other being in the same sort of building; you get to learn more about what therapies do, what nurses do in terms of the patients that they see, some of the stuff that's out there in terms of support for patients (Service manager).

However, both social care and health professionals mentioned a limited understanding in each other's roles and responsibilities. For example, social workers mentioned that community nurses lacked sufficient knowledge of the Care Act (Richardson, 2013), which resulted in nurses proposing care plans that were unworkable as they did not take into account the Local Authority parameters for care packages. Both groups of professionals suggested that these issues be could be partly mitigated by co‐location which would provide more opportunities for case discussion and allocating tasks.

One interviewee attributed the misunderstanding of roles and responsibilities to the increasingly specialist approach to healthcare, suggesting that a lack of generalists resulted in a quite narrow approach to care provision and an inability to retain a wider appreciation of the needs of patients.

#### 3.3.3 Developing working and social relationships

Interviewees provided contrasting perspectives of whether co‐location had fostered the development of more effective working and social relationships between different professional groups. Middle managers suggested that co‐location would foster relationships based on the premise that the sharing of office space would breakdown professional barriers. The development of working relationships appeared to be context dependant and varied among the teams. In one team, continuity of leadership and staff within the team was key to developing a positive culture. The team lead was an advocateof of flat hierarchies, promoted autonomy in decision making and encouraged staff to pursue training and professional development opportunities. In this team, co‐location of health professionals over a period of years had enabled the development of long‐standing social relationships which fostered more effective team working.

The xxx team are an exemplar for co‐location. All health professionals are located in the same open plan office with the team lead. The team have been together for a while and there is a solid foundation for effective communication and partnership working. Some of the team members tell me they enjoy this set up – it enables good communication – they can discuss patients easily. They seem to have built excellent social relationships too. There is a really harmony about the team that I have yet to observe elsewhere in the borough (ML field notes).

A lack of continuity of permanent staff and the reliance upon agency workers especially in social care was seen as barrier to partnership working. Permanent staff expressed frustration about the reliance upon agency workers citing this as a barrier to care continuity. At the time of the study, health professionals in the EPCTs were undergoing training to adapt working practices with a greater focus on providing more holistic care while reducing task orientated activities such as the administration of eye drops and insulin. However, agency staff in the EPCTs were seen as less likely to embrace partnership working; preferring to operate in their professional silo, work within the parameters of their role and be less willing to employ a holistic approach (more inclined to be task orientated in care delivery). EPCT staff also highlighted that the continual turnover in social workers hindered the development of effective working relationships.

You go into the nurse's office and there are new faces every other week, there is no continuity of care for patients. And to be fair, I don't think the agency staff have a chance to be properly inducted, they don't know about how we do things here. So they will see a patient but they won't have an idea about the services available locally or in fact what other support they can call upon from the team like the care navigators who can sort out lots of non‐medical issues (EPCT team member).

In two of the EPCTs, it was suggested that co‐location had not been an effective enabler in developing relationships. Some EPCT staff mentioned that professional identity characterised by the officious approach of some senior nurses in the EPCTs, acted as a barrier to forming relationships. This finding was consistent with similar opinions expressed by social workers who deemed the culture in the EPCTs as being quite hierarchical with some senior community nurses behaving as though social care was the ‘inferior’ partner.

## 4 DISCUSSION

The study findings show that, due to its multifaceted nature, co‐location alone is not an effective enabler for partnership working and, hence, should not be seen as a silver bullet for service integration. Co‐location provides a basis for joint working, but organisations must not overlook the various challenges we have identified in this study. In particular, professional identity, limited understanding of roles and responsibilities and a lack of continuity in personnel are significant barriers to service integration and partnership working (Lalani et al., 2020). Our finding that co‐location increases the frequency of informal and formal discussions between health professionals is similar to those reported elsewhere. Health professionals working in close proximity will have more frequent informal interactions than those who are geographically separated (Seaton et al., 2020). Corridor conversations and staff room discussions have also been shown to contribute to service improvement (Liberati et al., 2019).

For service integration to be effective, it requires joint working with social care. The power imbalance between health and social care has been a prominent issue for several years (Leutz, 1999). Hence, a key aim of the Vanguard programmes was closer alignment of health and social care at all levels of the care system which has been realised in part, at the strategic level by the development of new governance, managerial and administrative structures and joint commissioning (Glasby & Miller, 2020). Yet, our findings suggest that at the service delivery level, integration across health and social care has yet to be achieved. In this study, social workers were allocated workspace in the EPCT offices, but the quality of this space, a lack of understanding of social care provision on part of the nurses and a reliance on agency workers (especially in social care) acted as barriers to service integration. Furthermore, the stringent restrictions on the availability of NHS data mean that social workers are reliant on health colleagues verbally sharing information about patients/clients. A shared care record ought to be prioritised by the NHS executive bodies with access provided for social care to patient/client records and vice versa.

Despite the merits of co‐location and organisations working to address some of the relational aspects we have cited here, health system factors are likely to act as barriers to service integration. The lack of permanent staff in both health and social care affects the continuity of care, impairs the development of working and social relationships and undermines new care practices. These issues are exemplified in this study by agency nurses carrying out task‐orientated activities somewhat undermining the holistic approach pursued by permanent staff. Moreover, the constant turnover of staff in social care and reliance upon agency workers hampered communication and may undermine efforts to develop relationships across care boundaries. Such issues may also result in poorer patient/client outcomes. For example, locum doctors have been perceived as presenting a greater risk of causing harm to patients and similar reports have been published about agency nurses, although the evidence for an adverse effect on patient safety is not conclusive (Ferguson & Walshe, 2019; Page, 2008).

The lack of continuity of social workers or irregular attendance at multidisciplinary meetings hinders collaborative practice. Moreover, the absence of a voice for social care in discussions associated with the development of local integrated care services risks an imbalance in the delivery of services, weighted in the favour of healthcare (Bussu & Marshall, 2020; Lalani et al., 2020).

Co‐location may also be affected by the frequency and length of time with which staff are present in the office. Increasing numbers of patients with highly complex care needs whose care is gradually being prioritised in the home setting results in greater workloads for community care professionals with staff spending much of their working day away from the office (RCN, 2019). Digitisation and technological advancement including the drive for more remote working especially in the context of the Covid‐19 pandemic provide new challenges for partnership working. Coupled with the pervasive problem of a health and social care workforce gap, provider organisations will have to consider how these challenges will impact upon effective partnership working and how services are best organised and delivered with these challenges in mind.

This study has important implications for practice and research. Firstly, at the service delivery level, health and social care organisations need to be bolder and situate social workers with community health care teams, providing equity in the working environment and considering co‐funding the employment of community social workers whose primary role would be managing the social care provision for patients/clients on community health team lists. Secondly, given the increasing trend toward service integration and co‐locating of different professional groups strengthened by the recent mandating of Primary Care Networks in England (Wilson & Lewis, 2019), organisations must invest in organisational development activities (Bussu & Marshall, 2020). Taking proactive approaches to address known issues of professional identity through the provision of joint training, opportunities for networking, formal meetings and informal social events would optimise co‐location efforts. This should include consensus building and the promotion of shared values and beliefs or at the very least, an understanding and respect for differences in professional approaches such as decision‐making and organisational requirements. Finally, future research should aim to establish the effects of co‐location on health service and patient outcomes. In this study, co‐location was thought to reduce duplication of care provision as well as reduce the time interval between identifying an issue in care and addressing it.

### 4.1 Strengths and limitations

A limitation of the study is that findings are drawn from interviews with four multi‐professional teams in a borough of East London; hence, they may not be regarded as representative of co‐located teams in health and social care. Although we only interviewed a small proportion of all EPCT staff, we purposively selected participants based on profession and qualification status so as to obtain a representative view from the EPCTs. Moreover, our findings could be transferable as interviews were also held with a range of social care professionals including service and middle managers from both sectors. It is accepted that the patient/user voice is absent from this study. Their inclusion may have provided an understanding of quality of care outcomes (patient experience and satisfaction) as well as their perspectives identifying the effectiveness of co‐location and service integration. A strength of this study is the in‐depth participatory approach which provided a nuanced understanding of the dynamics of co‐located teams.

## 5 CONCLUSION

Co‐location is an integral step in the development of multi‐professional teams and as part of local service integration arrangements but other conditions need to be met for it to be effective in promoting partnership working. Organisations considering embarking on co‐locating different professionals may want to consider investment in adequate facilities, local systems that facilitate information sharing, continuity of personnel and the promotion of organisational development activities. The availability of resources and funding coupled with strategic alignment between health and social care will support integration but do not guarantee effective partnership working at the frontline. Organisations that are committed to greying care boundaries and providing joined up patient care must ensure that sufficient focus is provided at the service delivery level and not assume that decades of silo working in health and social care and strong professional cultures will be resolved by co‐location.

## CONFLICT OF INTEREST

No competing interests are declared.

## AUTHOR CONTRIBUTIONS

ML and MM were involved in the conception and design of the study. ML undertook data collection and analysis. All authors were involved in the process of interpreting the data. ML prepared the manuscript. All authors have read and approved the content of the final version of the manuscript.

## Data Availability

The datasets generated and/or analysed during the current study are not publicly available. This is due to the participatory approach to the study with the data potentially containing information that could compromise the research participants.
